# Impulse control and correlation to dopamine agonist serum concentrations in people with Parkinson's disease

**DOI:** 10.1007/s00415-024-12870-8

**Published:** 2025-01-15

**Authors:** Sara C. Staubo, Ole Martin Fuskevåg, Mathias Toft, Ingeborg H. Lie, Kirsti M. J. Alvik, Pål Jostad, Stein H. Tingvoll, Hallvard Lilleng, Kristina Rosqvist, Elisabet Størset, Per Odin, Espen Dietrichs, Erik Sveberg Dietrichs

**Affiliations:** 1 Department of Neurology, Oslo University Hospital, Rikshospitalet, Nydalen, PO Box 4950, N-0424 Oslo, Norway; 2 Center for Psychopharmacology, Diakonhjemmet Hospital, Oslo, Norway; 3 Institute of Oral Biology, University of Oslo, Oslo, Norway; 4 Institute of Clinical Medicine, University of Oslo, Oslo, Norway; 5 Experimental and Clinical Pharmacology, Institute of Medical Biology, UiT The Arctic University of Norway, Tromsø, Norway; 6 Department of Laboratory Medicine, Division of Diagnostic Services, University Hospital of Northern Norway, Tromsø, Norway; 7 Division of Neurology, Department of Clinical Sciences, Lund University, Skåne University Hospital, Lund, Sweden; 8 Department of Neurology, University Hospital of Northern Norway, Tromsø, Norway; 9 Unicare Fram Rehabilitation Centre, Rykkinn, Norway; 10 Ringen Rehabilitation Centre, Moelv, Norway; 11 Department of Neurology, Akershus University Hospital, Nordbyhagen, Norway; 12 Department of Clinical Medicine, UiT The Arctic University of Norway, Tromsø, Norway

**Keywords:** Parkinson’s disease, Pharmacology, Dopamine agonist, Impulse control disorder

## Abstract

**Background:**

Impaired impulse control is often seen in Parkinson’s disease (PD) patients using dopamine agonists.

**Methods:**

We performed a therapeutic drug monitoring study of 100 PD patients using ropinirole or pramipexole extended release. Three blood samples were collected on the same day. Serum concentrations were measured, and 24 h area under the curve (AUC) calculated. The validated Questionnaire for Impulsive-Compulsive Disorders in Parkinson's Disease-Rating Scale (QUIP-RS) was used for assessing impulse control.

**Results:**

Total ropinirole drug exposure showed weak, but significant correlation to the QUIP-RS score. No correlation between pramipexole serum concentrations and QUIP-RS was found. In ropinirole patients, both agonist dose and total dopaminergic treatment were correlated with QUIP-RS. Duration of ropinirole treatment correlated with impaired impulse control, and duration of dopaminergic treatment of any type correlated with QUIP-RS scores in both ropinirole and pramipexole patients.

**Conclusions:**

Our main finding is that impaired impulse control is correlated to both total drug exposure (AUC) and dopamine agonist dose for ropinirole, but not for pramipexole. These observations indicate that different strategies may be useful for treating PD patients with impaired impulse control: ropinirole dose reduction could be beneficial, whereas pramipexole treatment may have to be stopped.

## Introduction

Dopamine agonists are commonly used for symptomatic treatment of motor symptoms in Parkinson´s disease (PD). They are effective, but somewhat less potent than levodopa. The onset of motor complications like wearing-off and dyskinesias may be delayed when treatment is started with a dopamine agonist and levodopa used as add-on therapy [1, 2]. However, dopamine agonists are associated with more non-motor complications, and there is a considerable concern for the possible development of impulse control disorders (ICDs) [3–10].

Compulsive gambling, excessive buying, hypersexuality, and eating behaviors like binge-eating are usually defined as the four classical ICDs [11]. Several studies have confirmed the association between dopamine agonist use and ICDs in PD patients, but incidence and prevalence figures vary considerably among studies [3–10, 12–14]. Dopamine agonists have also been associated with other forms of reduced impulse control, such as dopamine dysregulation syndrome, punding and hobbyism [11]. When comparing previous reports, variable use of diagnostic tools and different ICD definitions represent a considerable problem. Currently, the only scale with validated cut-off values for defining the four classical ICDs is the Questionnaire for Impulsive-Compulsive Disorders in Parkinson's Disease-Rating Scale (QUIP-RS) [15]. While some of the studies have based their findings on this scale [16, 17], others have used clinical interviews [5, 6, 18–22], or other assessment questionnaires [7–10, 23–26]. Moreover, while early reports focused on single ICDs like hypersexuality [27, 28] or gambling [29], most of the more recent studies have included the four classical ICDs as well as other and less defined impulse control problems [9, 16, 18, 22].

There is a paucity of pharmacological studies exploring impulse control among PD patients treated with dopamine agonists. Previous research has examined the potential association between dopamine agonist dosage and impaired impulse control. Some studies have found a correlation [18, 19, 24, 25], others not [7, 23]. Two recent studies have examined dopamine agonist serum concentrations in PD patients and compared to impulse control behaviors[30, 31]. Contin et al. measured dopamine agonist serum concentrations at the assumed minimal concentration (C_min_), which is common practice in therapeutic drug monitoring (TDM). They found no correlation between serum concentrations of pramipexole, ropinirole, or rotigotine and ICDs and/or related behaviors [30].

We performed a detailed TDM multicenter study in Scandinavia, the ICD Parkinson Agonist Pharmacology Study (IPAPS). Altogether 100 PD patients using a morning dose of either pramipexole or ropinirole extended release were included. A higher number of participants as well as a higher number of recruiting centers were planned, but recruitment was hindered by the COVID pandemics and subsequent local health regulations limiting admittance to laboratories and outpatient clinics. Rotigotine users were not recruited for this study, as the risk for impaired impulse control is considerably less on rotigotine treatment [31]. Serum concentrations were measured immediately before and six and twelve hours after daily dopamine agonist intake, to obtain both C_min_ and C_max_-values as well as calculating area under the curve (AUC), for assessing total drug-exposure. The patients were interviewed, examined clinically and tested with the QUIP-RS and several other assessment forms.

The main aim of the IPAPS was to investigate whether reduced impulse control behavior correlates with pharmacokinetic measures, as addressed in this paper. In addition we chose to look separately at the twenty patients (10 using pramipexole, 10 on ropinirole) that scored above QUIP-RS cut-off for at least one ICD in a small, preliminary case–control study [32]. A correlation was found between the assumed maximal ropinirole serum concentration (C_max_), daily dose and ICD diagnosis, but this was not found for pramipexole.

In this main investigation, we have included all 100 participants to explore possible correlations between dopamine agonist serum concentrations and any degree of impaired impulse control behavior. We used the QUIP-RS, which is the only scale validated for evaluating the level of impulse control [15].

## Methods

### IPAPS

All 100 patients participating in the IPAPS have been included in this study. The IPAPS is a clinical and pharmacological multicenter study with a cross-sectional observational study design. We recruited patients during the period from spring 2020 to fall 2022. Patients were included from 4 centers in Norway (Oslo University Hospital, University Hospital of Northern Norway, Ringen Rehabilitation Center and Unicare Fram Rehabilitation Center) and one center in Sweden (Skåne University Hospital, Lund). Demographic data are given in Table 1. Further details have been provided in our IPAPS case–control article [32].

**Table 1:** Demographic data

Characteristics	Overall			Ropinirole users			Pramipexole users		
Number of participants	100			66			34		
Gender	56 male; 44 female			37 male; 29 female			19 male; 15 female		

	Mean	Median	Range	Mean	Median	Range	Mean	Median	Range
Weight (kg)	80.7	80.0	48–120	81.9	80.5	48–120	79.2	78.2	61–110
Height (cm)	175.0	177.0	148–195	174.8	176.5	156–195	175.4	177.0	148–194
Time since symptom debut (months)	104.8	94.0	15–276	107.2	98.0	15–276	100.0	81.0	32–217
Time since PD-diagnosis (months)	81.9	71.5	5–264	82.8	80.0	5–264	80.2	60.0	21–190
Time with dopaminergic treatment (months)	72.3	60.0	3–264	74.1	63.0	3–264	69.0	53.0	6–184
Time with dopamine agonist treatment (months)	69.9	60.0	1–252	68.5	60.0	1–252	72.8	60.0	18–184
Total LEDD	745.1	744.5	40–1627	731.4	716	40–1474	771.9	769.9	159–1627
LEDD dopamine agonist	216.8	200.0	36.4–480	205.4	200.0	40–480	239.0	219.8	36–441
Creatinine (μmol/L)	74.2	72.5	40–134	73.1	69.0	40–114	75.6	74.0	46–134
Hoehn and Yahr stage	2.0	2.0	1–4	2.0	2.0	1–4	1.9	2.0	1–4
MDS-UPDRS III	18.9	16.0	5–61	19.9	16	6–61	16.9	15.5	5–40
MDS-UPDRS IV	2.7	2	0–18	3.2	2	0–18	1.7	0	0–8
PDQ39-SI	35.5	33	20–67	35.8	33	20–67	34.8	32	20–60
NMSQ	9.4	9	0–21	9.3	8.5	0–21	9.5	10	1–19

Demographic data for all participants, and in the two right columns divided into participants using ropinirole and pramipexole. LEDD, levodopa equivalent daily dose; MDS-UPDRS, Movement disorders Society-Unified Parkinson’s Disease Rating Scale; NMSQ, Non-Motor Symptoms Questionnaire; PD, Parkinson’s Disease; PDQ39-SI, Parkinson's Disease Questionnaire 39-Summary Index

### Patients

Eligible patients had a diagnosis of idiopathic PD as defined by the International Parkinson and Movement Disorder Society clinical diagnostic criteria [33]. We included patients that used either pramipexole or ropinirole extended release in the morning, and that had not changed their dopaminergic medication during the last month. Patients were allowed to use other antiparkinsonian therapies. Inclusion and participation involved detailed clinical interviews, completion of study forms, clinical examination, and three blood tests within a twelve-hour period. Only patients who showed no signs of cognitive impairment during interviews were included.

### Standard protocol approvals, registrations, and patient consents

The study was approved by the Personvernombudet/Datatilsynet (General Data Protection Regulation in Norway; reference: 2018/6255), the Regional Ethical Committee in Northern Norway (reference: 2018/1343/REK nord), and the Swedish Ethical Board (reference: 2022-01340-01). Written informed consent was obtained from each study participant before inclusion.

### Impulse control score and correlation study

Patients were included whether they had experienced impaired impulse control or not. To assess the level of impulse control, we used the QUIP-RS total score and the separate subscores A-G for gambling, sexual behavior, buying, eating, hobbyism, punding and PD medication use, respectively [15]. The patients were asked to report their experiences from the last four weeks before examination. We also included two of the other most used scales for assessing impulse control: The Impulse control disorders and related conditions (ICDRC) questionnaire [34], and questions 6 and 7 of Scales for Outcomes in Parkinson´s Disease—Psychiatric Complications (SCOPA-PC) [35].

All 100 patients completed the QUIP-RS, but most patients also completed the ICDRC (62%) and questions 6 and 7 from the SCOPA-PC (64%). We combined the results from all three forms to calculate a separate Impulse Control-Score with subscores to see whether these would be more sensitive measures than the QUIP-RS scores alone (See Online Resource, Table 1). Correlation analyses were made both with the QUIP-RS scores alone and with the Impulse Control-Score and subscores. Using the Impulse Control-Score and subscores did not give additional information compared to the results obtained by the validated QUIP-RS form (Online Resource, Table 2), and only QUIP-RS data have therefore been reported here.

**Table 2:** Pharmacokinetic data

	QUIP-RS Median: 7 Range: 0–53	QUIP-RS A1-A4 Median: 0 Range: 0–9	QUIP-RS B1-B4 Median: 1 Range: 0–15	QUIP-RS C1-C4 Median: 1 Range: 0–11	QUIP-RS D1-D4 Median: 0 Range: 0–16	QUIP-RS E–G Median: 2,5 Range: 0–27
A. *Ropinirole*						
T0	*r*: 0.24 *p*: 0.05	*r*: 0.16 *p*: 0.21	*r*: 0.15 *p*: 0.25	*r*: 0.11 *p*: 0.40	*r*: 0.08 *p*: 0.54	*r*: 0.18 *p*: 0.16
T6	*r*: 0.24 *p*: 0.06	*r*: 0.06 *p*: 0.67	*r*: 0.12 *p*: 0.35	*r*: 0.16 *p*: 0.19	*r*: 0.04 *p*: 0.73	*r*: −0.10 *p*: 0.44
T12	*r*: 0.22 *p*: 0.09	*r*: 0.02 *p*: 0.90	*r*: 0.02 *p*: 0.90	*r*: 0.12 *p*: 0.37	*r*: 0.32 *p*: 0.01	*r*: 0.19 *p*: 0.14
AUC	*r*: **0.27** *p*: **0.04**	*r*: 0.05 *p*: 0.72	*r*: 0.16 *p*: 0.24	*r*: −0.07 *p*: 0.61	*r*: 0.06 *p*: 0.65	*r*: −0.09 *p*: 0.49
Age (years) Median: 61 Range: 40–82	*r*: −0.07 *p*: 0.59	*r*: **0.28** *p*: **0.03**	*r*: −0.01 *p*: 0.92	*r*: −0.01 *p*: 0.96	*r*: 0.05 *p*: 0.71	*r*: −0.01 *p*: 0.94
Gender Male: 37 Female: 29	*p*: 0.80	*p*: 0.55	*p*: 0.29	*p*: 0.13	*p*: 0.13	*p*: 0.94
Total LEDD Median: 716 Range: 40–1747	***r*: 0.27** **p: 0.03**	**r: 0.28** **p: 0.02**	*r*: 0.18 *p*: 0.14	*r*: 0.19 *p*: 0.13	*r*: 0.18 *p*: 0.16	**r: 0.26** **p: 0.04**
LEDD dopamine agonist Median: 200 Range: 40–480	**r: 0.30** **p: 0.01**	*r*: 0.22 *p*: 0.08	*r*: 0.22 *p*: 0.08	**r: 0.27** **p: 0.03**	**r: 0.32** **p: 0.01**	*r*: 0.24 *p*: 0.06

	QUIP-RS Median: 5 Range: 0–57	QUIP-RS A1-A4 Median: 0 Range: 0–5	QUIP-RS B1-B4 Median: 1 Range: 0–13	QUIP-RS C1-C4 Median: 0 Range: 0–13	QUIP-RS D1-D4 Median: 0 Range: 0–12	QUIP-RS E–G Median: 1 Range: 0–28
A. *Pramipexole*						
T0	*r*: −0.05 *p*: 0.76	*r*: −0.18 *p*: 0.31	*r*: −0.05 *p*: 0.76	*r*: −0.02 *p*: 0.89	*r*: −0.12 *p*: 0.51	*r*: −0.12 *p*: 0.49
T6	*r*: 0.06 *p*: 0.74	*r*: −0.07 *p*: 0.67	*r*: 0.06 *p*: 0.74	*r*: 0.01 *p*: 0.97	*r*: 0.27 *p*: 0.13	*r*: −0.02 *p*: 0.90
T12	*r*: 0.06 *p*: 0.72	*r*: −0.08 *p*: 0.66	*r*: 0.03 *p*: 0.86	*r*: 0.03 *p*: 0.88	*r*: −0.09 *p*: 0.61	*r*: −0.02 *p*: 0.89
AUC	*r*: 0.03 *p*: 0.85	*r*: −0.09 *p*: 0.63	*r*: −0.01 *p*: 0.97	*r*: 0.01 *p*: 0.96	*r*: 0.27 *p*: 0.14	*r*: −0.04 *p*: 0.82
Age (years) Median: 64.5 Range: 42–87	*r*: 0.24 *p*: 0.16	*r*: −0.06 *p*: 0.74	*r*: −0.08 *p*: 0.65	*r*: 0.24 *p*: 0.16	**r: 0.45** **p: < 0.01**	*r*: 0.23 *p*: 0.19
Gender Male: 19 Female: 15	*p*: 0.30	*p*: 0.10	*p*: **0.01**	*p*: **0.04**	*p*: 0.13	*p*: 0.81
Total LEDD Median: 716 Range: 159.2–1627	*r*: 0.11 *p*: 0.52	*r*: 0.14 *p*: 0.43	*r*: 0.21 *p*: 0.23	*r*: 0.07 *p*: 0.69	*r*: < 0.01 *p*: 1.00	*r*: 0.10 *p*: 0.58
LEDD dopamine agonist Median: 200 Range: 36.4–441	*r*: −0.03 *p*: 0.87	*r*: 0.02 *p*: 0.90	*r*: 0.12 *p*: 0.49	*r*: 0.02 *p*: 0.90	*r*: 0.08 *p*: 0.65	*r*: −0.17 *p*: 0.33

Pharmacokinetic data for **A** ropinirole and **B** pramipexole participants. Significant values are bold

### Covariates

All enrolled participants underwent a comprehensive neurological examination, which included a thorough assessment of motor function. Scores were made using the MDS-Unified Parkinson's Disease Rating Scale (MDS-UPDRS) parts III (ON medication) and IV [36], and the Hoehn and Yahr staging scale [37]. The examiner categorized whether PD was tremor dominant, rigid-akinetic, or mixed. During interviews patients were asked to provide information about the onset of PD symptoms, time of diagnosis, treatment history, comorbidities and possible problems related to impaired impulse control. All dopaminergic treatments were converted to levodopa equivalent daily dose (LEDD) [38]. In addition, the participants completed the Non-Motor Symptoms Questionnaire (NMSQ) [39], and PD specific health-related quality of life was assessed by the Parkinson's Disease Questionnaire (PDQ-39) [40].

### Blood tests and pharmacological analyses

We collected three blood tests from each participant on the same day. The first sample was obtained in the morning, just before their daily dose of dopamine agonist, representing C_min_. The second sample was taken 6 h later, corresponding to the assumed time (T_max_) it takes to reach maximal serum concentration (C_max_) after intake. Both ropinirole and pramipexole extended release formulations have T_max_ of approximately 6 h. Finally, a third sample was collected after 12 h to calculate the area under the curve (AUC) from 0 to 24 h (AUC0-24 h). Following collection, the blood samples were centrifuged, plasma was extracted and directly frozen. These frozen samples were then sent to the University Hospital in Northern Norway for analysis.

Some of the samples were missing from two patients (one in the pramipexole and one in the ropinirole group). Data from these two patients were omitted from the pharmacokinetic analyses.

We measured ropinirole and pramipexole in serum with a validated method using liquid chromatography connected to a tandem mass spectrometer (LC–MS/MS). UniSpray was utilized for ionization. Sample preparation involved liquid–liquid extraction of analytes, along with the use of isotope-labeled ropinirole and pramipexole as internal standards to minimize matrix effects. Detailed information about the analytical procedures was published as supplementary material to our IPAPS case–control article [32].

### Area under the curve calculation and statistical analyses

We assessed the AUC for each patient over a 24-h period. The AUC0-24 h was calculated using a non-compartmental analysis based on samples at 0 h (C_min_), 6 h and 12 h. We did not directly measure the concentration at 24 h but assumed that the predose sample at 0 h was equivalent to the sample at 24 h. Additionally, we relied on steady-state pharmacokinetics because there was no change in dopaminergic medication during the last month before inclusion.

To calculate the AUC0-24 h, we used the linear up/log down trapezoidal method with the package “PK” in R v.4.2.1.

The data were found to not be normally distributed (by Henze–Zirkler Bivariate Normality Test) and accordingly the strength of association between QUIP-RS scores, the Impulse Control-Score and different treatment or patient data were investigated using the Spearman Rank Order Correlation in SigmaPlot version 14.5 (Germany). A correlation coefficient (r) approaching + 1 indicates a strong and positive relationship, considered significant if the p value was below 0.05. Differences in QUIP-RS score, dopamine agonist LEDD-score, total LEDD score and gender between the pramipexole and ropinirole treated patients were tested using Student’s t test if normality (Shapiro–Wilk) and equal variance tests (Brown–Forsythe) were passed. If not, a Mann–Whitney rank sum test was used instead.

## Results

Descriptive characteristics of the 100 included patients are given in Table 1. The groups of patients who used ropinirole and pramipexole were similar in terms of gender distribution, age, weight and height. There were no differences between ropinirole and pramipexole treated patients in QUIP-RS score (*p* = 0.97), total LEDD (*p* = 0.73) or dopamine agonist LEDD (*p* = 0.51).

### Serum concentrations

**Fig. 1:**
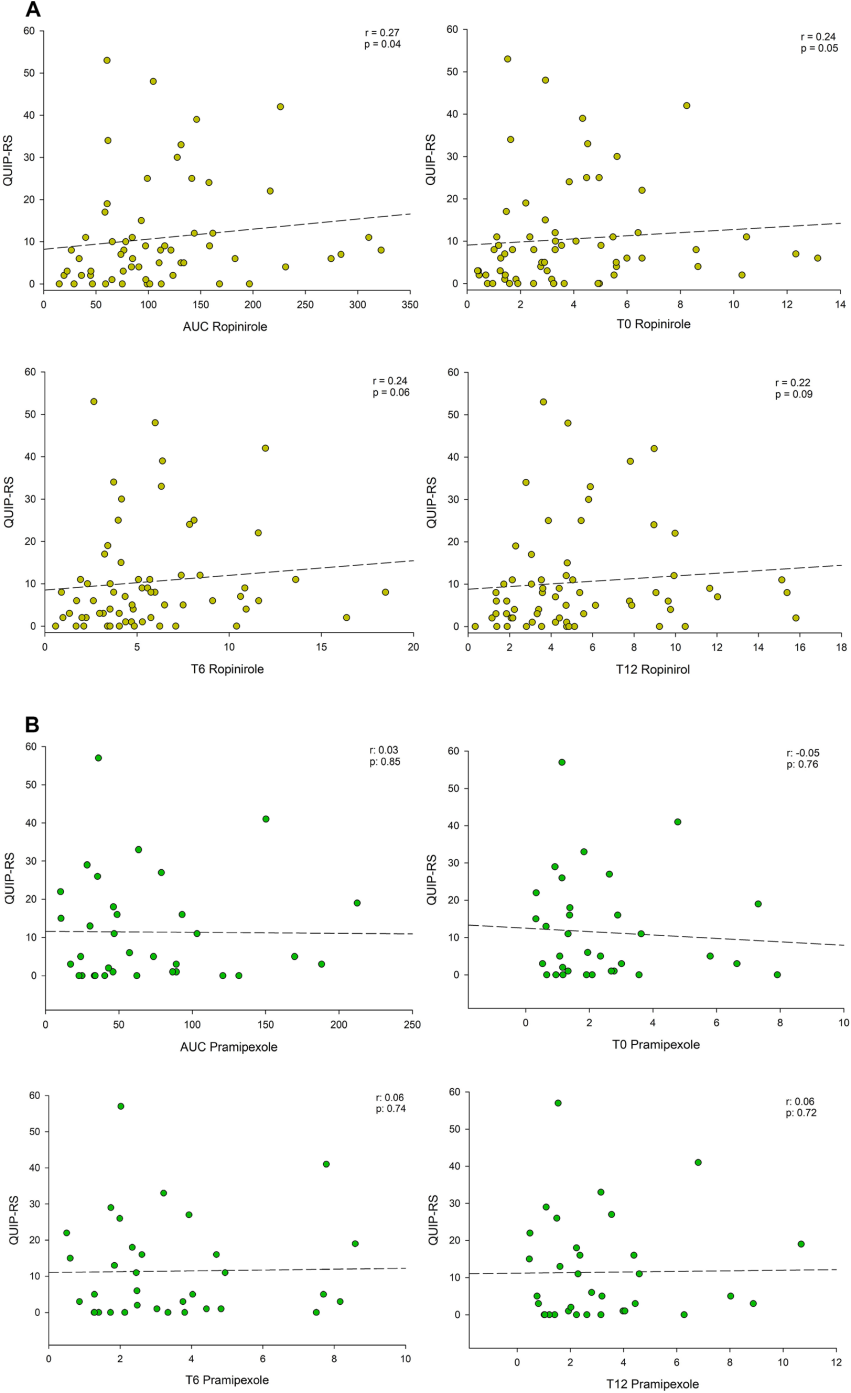
Dopamine agonist serum concentrations and impulse control. Correlation diagrams comparing serum concentration of **A** ropinirole and **B** pramipexole with QUIP-RS. T0 is the serum concentration just before daily dose intake, T6 is after 6 h and T12 is after 12 h. Area under the curve (AUC_0-24h_) was calculated assuming T24 = T0

AUC of ropinirole, indicating total drug exposure, correlated significantly with higher QUIP-RS scores. T0 concentrations, which is the standard test used for therapeutic drug monitoring, had a correlation p-value of 0.05 with QUIP-RS in ropinirole patients. No correlation was found between QUIP-RS and pharmacokinetic data in pramipexol treated patients. Details are shown in Table 2 and Fig. 1.

### Drug treatment

**Fig. 2:**
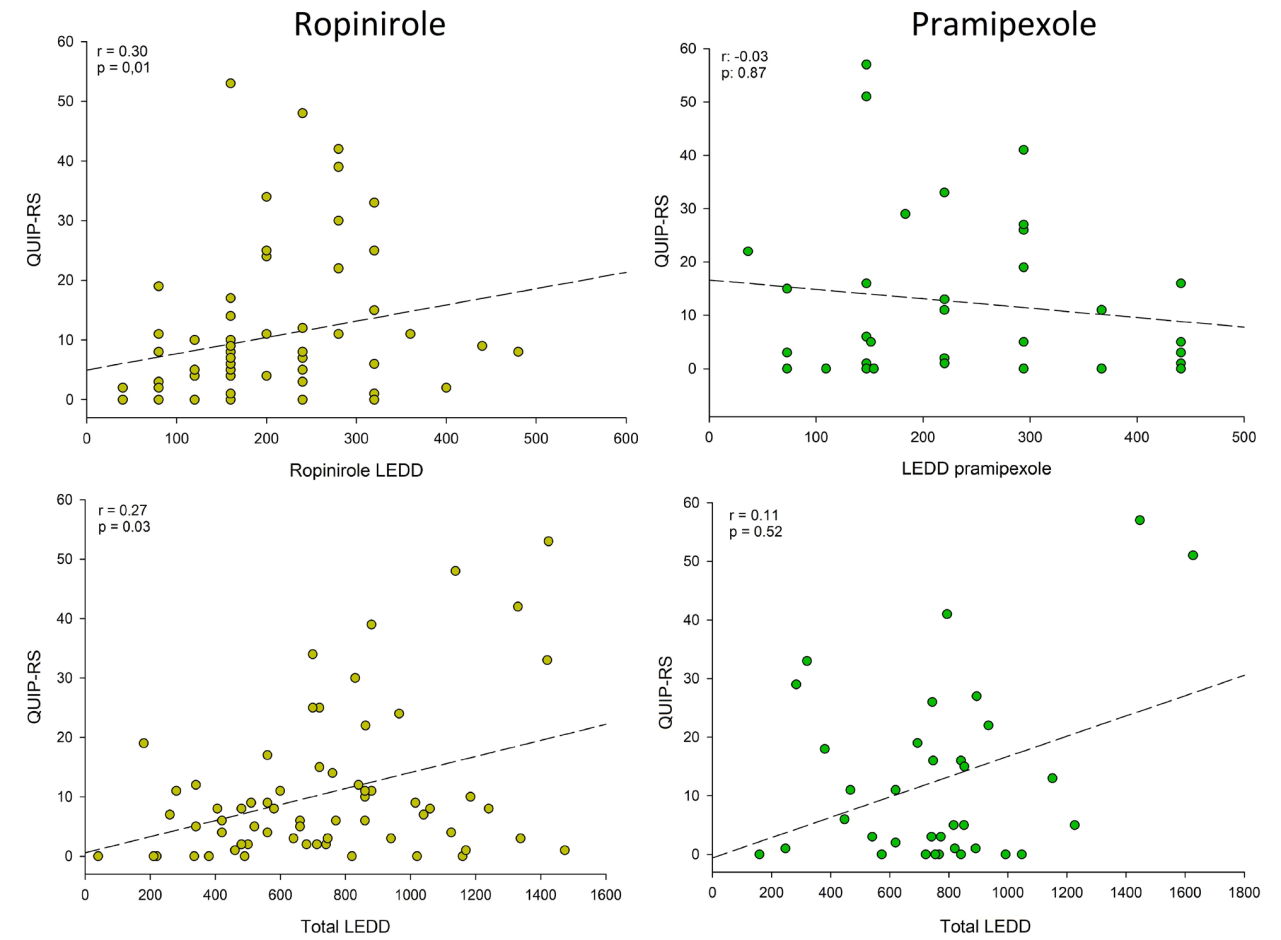
Dopamine agonist dose and impulse control. Correlation diagrams comparing LEDD dopamine agonist (dopamine agonist dose) and total LEDD with QUIP-RS. Results from the ropinirole group to the left, pramipexole to the right. LEDD, levodopa equivalent daily dose

In ropinirole-users we found statistically significant correlations between total LEDD and QUIP-RS score. There was also a correlation between ropinirole dose (LEDD ropinirole) and total QUIP-RS, and correlations between ropinirole dose and QUIP-RS subscores C and D (buying and eating) (Table 2A, Fig. 2). No such correlations were seen among the pramipexole users (Table 2B, Fig. 2).

### Duration of PD and treatment

**Fig. 3:**
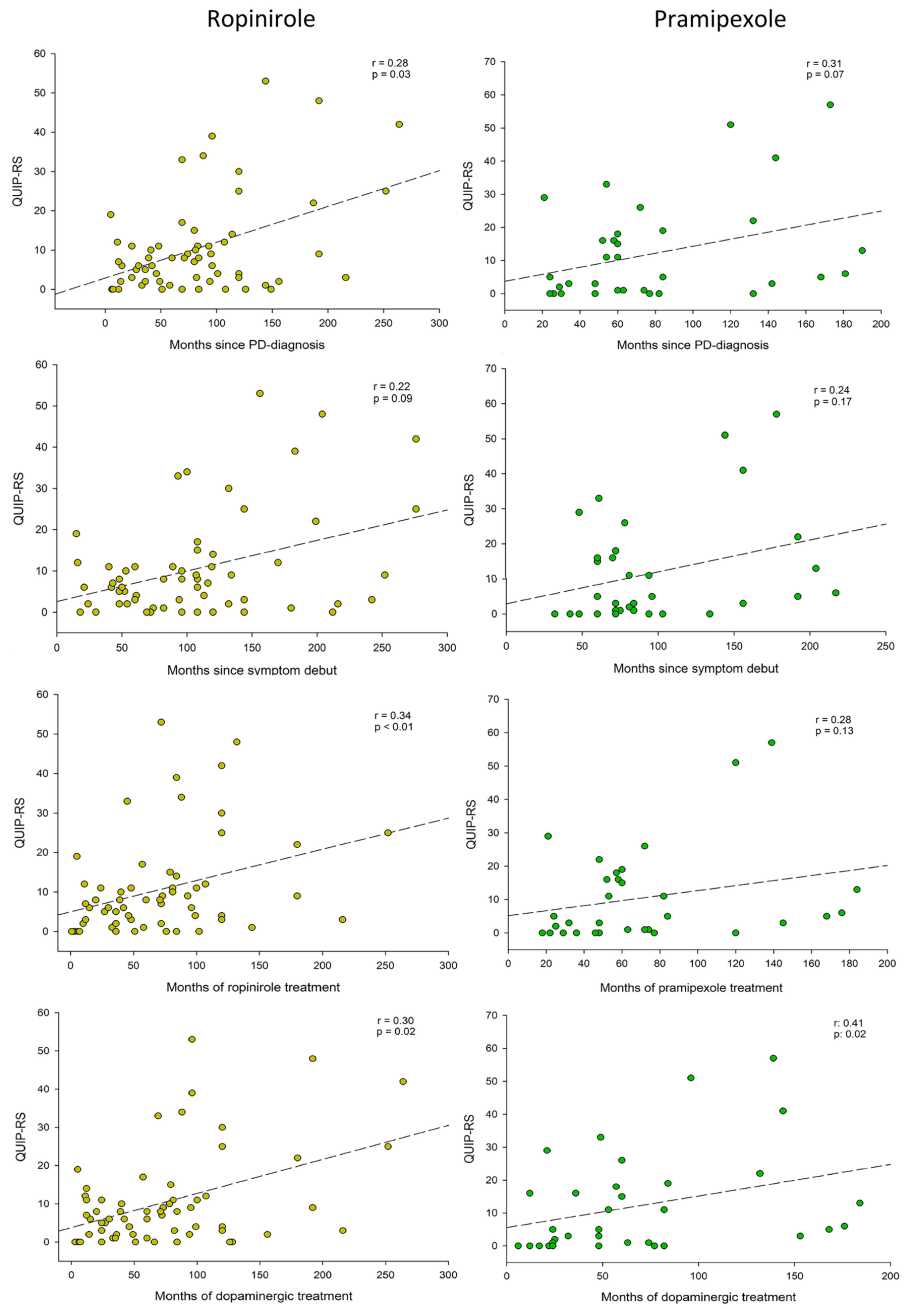
Impulse control in relation to the duration of disease and treatment. Correlation diagrams comparing months since PD diagnosis, months since symptom debut, months of dopamine agonist treatment and months of dopaminergic treatment with QUIP-RS

In the ropinirole group correlations were observed between the time since PD diagnosis, duration of dopaminergic treatment and duration of ropinirole treatment, and the QUIP RS score. For pramipexole there was only a significant correlation with duration of dopaminergic treatment compared to QUIP-RS (Fig. 3).

## Discussion

Our main finding is a significant, though weak correlation between total drug exposure (AUC) of ropinirole, calculated by serum concentration measurements throughout a day, and the QUIP-RS score. No correlation between pramipexole concentrations and QUIP-RS was found. To our knowledge, only two previous studies have examined the potential association between dopamine agonist serum concentrations and impaired impulse control behavior. Our current findings are consistent with a previous pharmacological study that found similar plasma levels at C_min_ for the three dopamine agonists pramipexole, ropinirole and rotigotine in patients with and without ICD and/or related behaviors, but the AUC was not calculated in that study [30]. In our small previous case–control study we found higher serum concentrations at assumed ropinirole C_max_ in patients scoring above cut-off for at least one ICD [32], but no associations between serum concentrations and ICD diagnosis for patients using pramipexole.

The present study has assessed correlation between impulse control and a range of clinical variables. Previous investigations have used several different methods for identifying patients with impulse control problems (see Introduction). We have based our results on QUIP-RS [15]. We also tried to use a combined Impulse Control-Score that in addition included scores from the ICDRC [34] and questions 6 and 7 from SCOPA-PC [35], but the results did not differ from those obtained with QUIP-RS alone. All patients also went through detailed clinical interviews. Compared to our own personal observations and the reports from caregivers and health professionals, it was obvious that many patients underreported their impulse control problems. All this taken together, we deemed the validated QUIP-RS to be the most reliable impulse control measure.

Pramipexole and ropinirole are usually considered the two dopamine agonists with highest ICD risk [8, 31]. For both agonists, we found that the risk for impaired impulse control increased with duration of dopaminergic treatment. A direct association between level of drug exposure and ICD was, however, only found for ropinirole, as our data shows no significant correlation between pramipexole serum concentration nor pramipexole dose (LEDD pramipexole) and QUIP-RS. In addition to total QUIP-RS, the ropinirole dose (LEDD ropinirole) had significant correlations with the subscores for buying and eating. In our case–control analysis [32], ropinirole C_max_ was significantly higher in patients diagnosed with ICD, and in the present study we found that the total drug exposure during a day (AUC) correlated with total QUIP-RS score. Therefore, it seems that variation in ropinirole pharmacokinetics is more important for impulse control than pramipexole pharmacokinetics, even though correlations in our material were found to be weak for both tested dopamine agonists. Correlations were weak also for total dopaminergic treatment, disease duration and disease severity. The weak strength of these correlations seems to indicate that other and hitherto unidentified factors may also be important for impaired impulse control.

Given that the amount of drug exposure may be important for impulse control in ropinirole patients, impaired impulse control cannot solely be caused by genetic susceptibility, environmental or disease-related factors that leave some individuals vulnerable for impulse control problems as an adverse effect of ropinirole. In the present study, we have only measured ropinirole, not its metabolites. It is possible that the risk of impaired impulse control is not increased by ropinirole itself but one of its metabolites. Ropinirole is primarily metabolized by the hepatic CYP1A2-enzyme [41]. CYP1A2-function is not subject to large genetical variations but could vary with age, disease and to a large extent—smoking. As smoking induces CYP1A2 and, therefore, reduce ropinirole serum concentrations, sudden cessation of smoking can increase serum-concentrations with adverse effects of ropinirole treatment [42]. Unfortunately, smoking history among our patients was not systematically registered. To speculate, heterogenous CYP1A2 metabolism of ropinirole between the patients in our study could also give varying serum concentrations of an unknown ropinirole metabolite inducing impulse control impairment.

A recently published expert consensus about the management of ICD and related disorders in PD recommended dose reduction of dopamine agonists as the initial step [43]. Our findings support that this strategy may be useful for ropinirole, but it is more uncertain whether pramipexole dose reduction is effective, and pramipexole may have to be stopped. Anyway, reduction of dopamine agonist treatment should be done with caution because of the withdrawal syndrome risk.

The underlying pathophysiological mechanism causing impaired impulse control in PD patients is not fully understood. The relative sparing of neurodegeneration of the medial dopaminergic neurons in the substantia nigra, and subsequently a preserved connectivity between the substantia nigra and the limbic system may be part of the underlying mechanism [44]. Especially stimulation of mesolimbic D3 receptors and pulsatile dopaminergic stimulation have been thought to play a role. It has been speculated that stimulation of D3 receptors is more associated with manifest ICDs, and pulsatile dopamine stimulation associated with punding and dopamine dysregulation syndrome [43, 45–47]. However, such differences are not obvious from our results. Pramipexole and ropinirole both act as D3 agonists. Our data show that total LEDD is correlated with higher total QUIP-RS score in ropinirole, but not pramipexole patients. Fifty-four of our ropinirole and 29 of our pramipexole patients used levodopa. None of them were on pump treatment. This means that most of our patients received pulsatile dopaminergic stimulation. In our analyses, we found no differences between total QUIP-RS score and subscores for punding and dopamine dysregulation correlated to total LEDD in pramipexole patients, but a correlation with punding in the ropinirole group. Also, the dopamine agonist cabergoline does not bind to D3 receptors, but is still associated with ICD development [48].

Individual vulnerability seems to be important for development of ICD. An increasing amount of evidence suggests that genetic polymorphisms in dopamine receptors could be important risk factors for ICD-development. Calculation of a dopamine genetic risk score has been suggested, based on investigation of known mutations in D1, D2 and D3-receptors as well as dopamine transporter and catechol-O-methyltransferase genes [49, 50]. When applied to pramipexole, where we found no clear correlation between dosage or serum concentration to QUIP-RS score, genotyping could potentially predict who are at risk for developing ICD during treatment.

### Strength and weaknesses

The main strength of this study is that IPAPS is the first pharmacological TDM looking at dopamine agonist serum concentrations throughout the day in PD patients treated with a stable dose of long acting pramipexole or ropinirole. All patients have been interviewed, clinically examined, and have provided detailed anamnestic data. We chose to use the QUIP-RS as the measure for impaired impulse control, as this is the only available validated tool, and as we found that combining with other questionnaires did not provide additional information. A major weakness is the number of patients enrolled, which had to be reduced considerably due to the Covid pandemics. Future studies with more patients are needed to verify the results.

Another problem is that the pramipexole group is smaller than the ropinirole group. Otherwise, the groups were comparable (Table 1). The proportions of patients using dopamine agonist monotherapy were also similar (15% in the pramipexole group, 18% in the ropinirole group), and there were no significant differences in total LEDD, dopamine agonist LEDD or QUIP-RS total scores between the two groups.

Other weaknesses are the patients’ presumable underreporting of impulse control problems, and that we do not have information about metabolite serum-concentrations. Anamnestic information about smoking would also have been valuable. This does however not include other forms of nicotine-intake, as it is substances produced by smoking per se that induces CYP1A2 [51].

## Conclusion

Total ropinirole drug exposure (AUC) shows a weak, but significant positive correlation with the QUIP-RS score in people with PD, while there is no correlation between pramipexole serum concentrations and impaired impulse control. The dose of pramipexole does not seem to be of major importance for the development of impulse control problems, while higher ropinirole dose and serum concentrations increase these problems. For all patients, duration of dopaminergic treatment is correlated with reduced impulse control. These findings could implicate that different strategies are useful for treating PD patients with impaired impulse control using either pramipexole or ropinirole.

## Data Availability

The datasets used and analyzed during the current study are available from the corresponding author on reasonable request.
